# Time trends and heterogeneity in the disease burden of glaucoma, 1990-2017: a global analysis

**DOI:** 10.7189/jogh.09.020436

**Published:** 2019-12

**Authors:** Yichi Zhang, Guangming Jin, Min Fan, Yifan Lin, Xin Wen, Zijing Li, Peng Zeng, Danying Zheng, Yuqing Lan

**Affiliations:** 1 Department of Ophthalmology, Guangdong Provincial Key Laboratory of Malignant Tumor Epigenetics and Gene Regulation, Sun Yat-sen Memorial Hospital, Sun Yat-sen University, Guangzhou, China.; 2 State Key Laboratory of Ophthalmology, Zhongshan Ophthalmic Center, Sun Yat-sen University, Guangzhou, China; 3 Department of General Intensive Care Unit, The Third Affiliated Hospital of Sun Yat-sen University, Guangzhou, China; * These authors contributed equally to this work.; † Joint equal contributions.

## Abstract

**Background:**

To evaluate the disease burden of glaucoma in terms of disability-adjusted life years (DALY) and assess the contribution of risk factors to DALY due to glaucoma.

**Methods:**

Global, regional, and country DALY number, rate, and age-standardized rates of glaucoma were obtained from the Global Burden of Disease Study 2017 database. The Human Development Index (HDI), Inequality-Adjusted HDI, Socio-Demographic Index (SDI), and other country-level data were derived from international open databases. Regression analysis was used to assess the correlations between the age-standardized DALY rate and the variables.

**Results:**

The global DALY due to glaucoma increased by 81% from 1990 to 2017 and decreased by 10% over the last two decades after adjusting for age and population size. Males had higher age-standardized DALY rates (*P* < 0.001). The age-standardized DALY rate was higher in countries with lower income or lower SDI (*P* < 0.001). The country-level age-standardized DALY rates in 2017 were negatively associated with HDI, SDI, country-level age-standardized prevalence rates of cataracts, cataract surgery rates (CRS), physician rates, and Inequality-Adjusted HDI. Stepwise multiple regressions showed that HDI, CRS, and Inequality-Adjusted HDI were significantly negatively associated with the country-level age-standardized DALY rate in 2017 after adjusting for other confounding factors (*P* < 0.001).

**Conclusions:**

Higher education, higher CRS, and diminishing the inequality in resource distribution may help reduce the disease burden of glaucoma. These findings can provide information for policymakers and could serve as an impetus for efforts toward alleviating the disease burden of glaucoma.

The Global Burden of Disease Study 2017 revealed that with increasing population sizes, ageing populations, extended life expectancy, and falling death rates in most countries and territories, disorders that cause disability but not substantial mortality have assumed greater proportions in the burden of disease [1]. These are the results of the growing and ageing population and socioeconomic development, and disabling diseases are expected to become greater public health concerns in the coming decades [1]. Glaucoma is a disabling diseases that mostly targets the elderly [2]. It is the second leading cause of blindness and the leading cause of global irreversible blindness [3]. A recent study reported that 3.5% of the world's population, or 64.3 million people, have glaucoma. Approximately 5.7 million people are visually impaired, 3.1 million are blind, and the number of people with glaucoma worldwide may increase to 111.8 million in 2040 [3]. Unlike cataracts and refraction/ accommodation disorders, glaucoma is not yet curable; it causes irreversible visual impairment. It is an optic neuropathy characterised by progressive structural and functional changes of the optic nerve leading to irreversible damage if untreated [4,5]. Those with glaucoma-induced visual impairments generally suffer from decreased vision-related quality of life (including reduced vision-dependent mobility and increased incidence of falls) [6,7]. This places a substantial burden on medical care systems [8-10]. Glaucoma is often associated with a long and asymptomatic initial phase and usually progresses unnoticed until extensive and irreversible vision loss has occurred. In this stage, the effects of medical and surgical treatment can be unsatisfactory, underscoring the importance of the early detection and treatment of this disease. Once glaucoma has been diagnosed, it requires lifelong follow-up and management to prevent further loss of vision. The recognition of the magnitude and distribution of glaucoma’s disease burden is of pronounced importance to inform clinicians and researchers and will guide policymakers in health services allocation.

The disease burden can be measured by financial cost, mortality, and morbidity. It can be quantified by the disability-adjusted life years (DALY), which is defined as the sum of years of healthy life loss caused by ill health, disability, or early death [1]. DALY reflects the gap between the actual health status and the normative situation. The DALY is calculated by taking the sum of the years lost due to disability (YLD) and the years of life lost (YLL). The YLD measures the burden of living with any short-term or long-term health loss (a disease or disability). It is determined by the number of disabled years weighted by the level of disability caused by a disability or disease. The YLL due to premature mortality is the multiplication of deaths and a standard life expectancy at the age of death. When a condition did not result in an individual dying younger than expected, it was not counted. One DALY means 1 lost year of healthy life due to disease. The advantage of the DALY is it can be compared between different diseases and injuries across time and regions and can be analysed using socioeconomic indexes. The DALY’s change and distributions have been analysed for several disability diseases [11-17], but there are few studies for glaucoma. The most recent study of The Global Burden of Diseases, Injuries, and Risk Factors (GBD) demonstrated that vision loss was the third largest impairment in DALY after anaemia and hearing loss globally from 1990-2017. Of all vision-threatening disorders, glaucoma was the leading cause of irreversible vision loss, followed by age-related macular degeneration [1]. Given the positive association of glaucoma prevalence and advanced age, and the disease characteristics of irreversible vision loss and the requirement of life-long monitoring, glaucoma is expected to become an even greater public health concern in the future. The aim of this study was to evaluate the time trends of the global disease burden of glaucoma and its distribution across age, gender, and country-levels of health care and socioeconomic development.

## METHODS

### Disease burden of glaucoma

The DALY data due to glaucoma was obtained from the open database of the Global Burden of Disease 2017 Study in the GHDx (http://ghdx.healthdata.org/gbd-results-tool), which provided an index to estimate the disease burden of more than 328 diseases and injuries in 195 countries from 1990 to 2017. The methodology of the Global Burden of Disease 2017 Study was detailed in a previous publication [1]. In brief, DALY was calculated as the sum of the YLD and the YLL resulting from glaucoma vision loss. The YLD = the number of prevalent cases * the disability weight, and the YLL = the number of deaths × the standard life expectancy at age of death in years. However, due to its nonfatal nature, there are no YLLs attributed to glaucoma, so the DALY due to glaucoma equals the YLD in GDB 2017 [18-20]. To calculate the YLD, vision loss was divided into three levels according to the World Health Organisation (WHO): blindness (completely blind, which causes great difficulty with some daily activities, worry and anxiety, and problems leaving home without assistance), severe vision impairment (which causes difficulty with daily activities, some emotional impact, and problems leaving home without assistance), and moderate vision impairment (which causes vision problems that make it difficult to recognise faces or objects across a room). The disability weights were 0.187, 0.184, and 0.031, respectively. DALY due to glaucoma was calculated for populations aged 45 and older. The DALY rate was calculated by adjusting for population size (per 100 000 population), and the age-standardized DALY rate was obtained by further adjusting for population size and age structure (per 100 000 population).

The following data were obtained for statistical analysis: (1) the global DALY number, rate, age-standardized rate, and prevalence number in 1990-2017; (2) the DALY numbers and rates of different age groups and genders from individual countries in 2017 (3); the World Health Organisation (WHO) regional and the World Bank regional income level and Socio-Demographic Index (SDI) level regional DALY numbers, rate, and age-standardized DALY rates in 2017; and (4) the country-level DALY numbers, rates, and age-standardized DALY rates in 2017. Global maps were generated from a data visualisation tool available from the Global Health Data Exchange (GHDx) supported by the Institute for Health Metrics and Evaluation (IHME) (https://vizhub.healthdata.org/gbd-compare/).

### Country-level indicators

The exposure factors were the country-level demographic, socioeconomic, and health care indicators derived from the following well-known open databases. Cataract is the leading cause of blindness worldwide [21]. Both glaucoma and cataract are intrinsically correlated with age. Recently there has been growing evidence to support the fact that cataract surgery can be a helpful tool in glaucoma treatment by lowering intraocular pressure which is the only treatable risk factor for glaucoma [22], so we used the prevalence of cataract as an indicator, and cataract surgery rate as a predictor. Axial lengths are also thought to influence the prevalence of glaucoma [23,24], as longer axial length is a risk factor of primary open angle glaucoma and short axial length is a risk factor of primary close angle glaucoma. The axial length is also a key factor to influence the refractive error, so we used the prevalence of refraction disorders as an indicator. The country-level prevalence of cataract and refraction disorders in 2017 was obtained from the Global Burden of Disease 2017 Study as previously noted. The cataract surgery rate (CSR) was obtained from a previously published study [25]. The values of nearest year available were used in this study. The open database of the United Nations Development Programme (http://hdr.undp.org/en/data) was used to obtain country-levels of the number of medical doctors per 10 000 people (that refer to the most recent year available during 2007-2017). The country-levels of the Human Development Index (HDI) in 2017 were also obtained from the United Nations Development Programme’s database, which is a composite index of social and economic achievement. The HDI has four components: A life expectancy index, a Mean years of schooling index, an Expected years of schooling index, and an Income index. The HDI ranges from 0 to 1, with a higher value indicating a higher socioeconomic level. Countries were classified into four groups: low (HD<0.550), medium (0.550-0.699), high (0.700-0.799), and very high (0.800 or greater) HDI. In addition, an Inequality-adjusted HDI that adjusted the inequality in the distribution of the HDI within each country was also obtained from this database. The country-level Socio-demographic index (SDI) in 2017 was obtained from the open database of the GHDx (http://ghdx.healthdata.og/ ). The SDI is a composite measure of a country’s socio-demographic development. It is based on the average income per person, educational attainment, and total fertility rate (TFR). The SDI ranges from 0 to 1; 0 represents the lowest income per capita, lowest educational attainment, and highest TFR from 1970 to 2017, and 1 represents the highest income per capita, highest educational attainment, and lowest TFR. Countries were placed into quintiles (high SDI, middle-high SDI, middle SDI, middle-low SDI, and low SDI) based on their 2017 SDI value.

### Statistical analysis

The outcomes included the time, age, gender, and geographic distribution of DALY due to glaucoma, as well as the influence of DALY, prevalence of cataract and refraction and accommodation disorders, socioeconomic indicators, and health care indicators on DALY rates due to glaucoma. The Wilcoxon signed rank test was used to compare the gender differences in DALY number and rate for each age group in 2017 and the age-standardized DALY rate among four income-based country groups and five SDI-based country groups across time. Scatter plots were constructed to explore the relationships between variables and age-standardized DALY rates of glaucoma at a country-level. Linear regression analysis was used to explore the influence of indicators on the age-standardized DALY rates of glaucoma at a country-level. Multiple linear regression modelling was fitted using the stepwise approach with the significance level set at 0.05. A P value <0.05 was considered statistically significant, The Bonferroni correction was used for multiple comparisons. Statistical analyses were performed using SPSS software (SPSS, Inc.; Chicago, IL, USA; version 23.0). Figures were drawn using GraphPad Prism software (version 7.0e, GraphPad Software; San Diego, CA, USA).

## RESULTS

### Trends in the disease burden of glaucoma from 1990 to 2017

**Figure 1:**
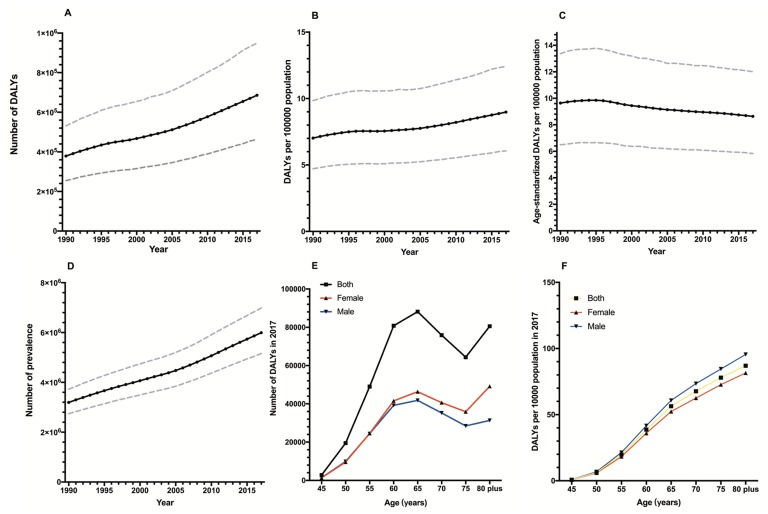
Time trends of the global disease burden of glaucoma and prevalence from 1990-2016 and distributions by age in 2016. **Panel A.** DALY numbers. **Panel B.** DALY rates. **Panel C.** Age-standardized DALY rates. **Panel D.** Prevalence of glaucoma. **Panel E.** DALY numbers in 2017. **Panel F.** DALY rates in 2017. Dashed lines represent 95% uncertainty intervals. DALY – disability-adjusted life years.

According to the GBD2017, the DALY number of glaucoma increased by 81%, from 378 970.01 (95% CI (confidence interval) = 254 589.52-531 355.36) in 1990 to 686 094.10 (95% CI = 462 923.89-948 844.26) in 2017 (**Figure 1****,** Panel A). The DALY rate increased by 28%, from 4.02 (95% CI = 7.02-8.97) to 6.24 (95% CI = 6.06-12.42) (**Figure 1**, Panel B), which eliminated the influence of population size. When the influence of both population size and age structure was eliminated, the age-standardized DALY rate of glaucoma increased up until 1995, and then decreased from 1995 to 2017. The global age-standardized DALY rate dropped from 9.64 (95% CI = 6.49-13.37) in 1990 to 8.64 (95% CI = 5.83-12.01) in 2017 and decreased 10% in 2017 compared to 1990 (**Figure 1****,** Panel C). In addition, the prevalence of glaucoma increased according to time (**Figure 1****,** Panel D).

### Global disease burden of glaucoma by age and gender

According to the GBD2017, **Table 1** shows the global DALY and prevalence of glaucoma in 2017. **Figure 1** shows the global DALY numbers and rates according to gender in different age groups in 2017. The two genders showed a similar trend in global DALY by age, sharply increasing before 60 years of age, then slowly rising to 65, and decreasing between 65 and 75, and increasing again after 75 years of age (**Figure 1****,** Panel D). After adjusting for the population size influence, the DALY rates of the two genders sharply increased according to age and the rate of males was higher than females, while the DALY number of males was lower than females (**Figure 1****,** Panel E). Wilcoxon signed rank tests showed there were significant gender disparities in the global DALY rate for each age group (*P* = 0.012), while no significant gender disparities were found in the global DALY numbers (*P* = 0.401).

**Table 1:** DALYs and prevalence of glaucoma in 2016

Gender	DALYs			Prevalence			
Value	95% CI upper	**95% CI lower**	**Value**	**95% CI upper**	**95% CI lower**	
**Number:**							
Male	342 235.06	473 645.49	230 353.06	2 972 930.22	3 471 931.16	2 557 883.95	
Female	343 859.04	478 347.14	232 756.09	3 020 577.15	3 530 927.46	2 596 361.48	
Both	686 094.10	948 844.27	462 923.89	5 993 507.37	6 984 803.24	5 157 462.27	
**Rate:**							
Male	8.93	12.35	6.01	77.53	90.54	66.71	
Female	9.03	12.57	6.12	79.36	92.77	68.22	
Both	8.98	12.42	6.06	78.44	91.42	67.50	
**Age-adjusted rate:**						
Male	9.38	12.92	6.35	81.66	94.90	70.31	
Female	8.03	11.16	5.43	70.52	82.43	60.60	
Both	8.64	12.01	5.83	75.57	88.09	64.97	

DALY – disability-adjusted life years, CI – confidence interval

### Global disease burden of glaucoma by income level and SDI level regions

**Figure 2:**
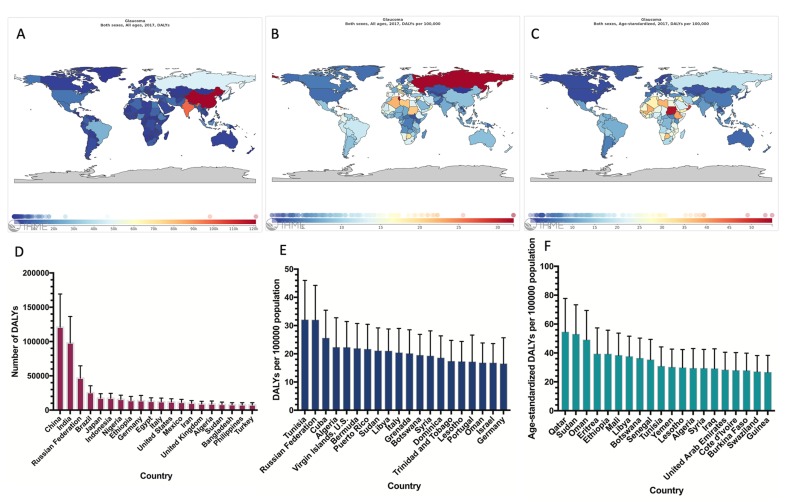
Global map of the disease burden of people visually impaired from glaucoma and the 20 countries with the highest burden. **Panel A.** DALY numbers. **Panel B.** DALY rates. **Panel C.** Age-standardized DALY rates. **Panel D.** The 20 countries with the highest DALY numbers. **Panel E.** The 20 countries with the highest DALY rates. **Panel F.** The 20 countries with the highest age-standardized DALY rates. DALY – disability-adjusted life years.

**Figure 3:**
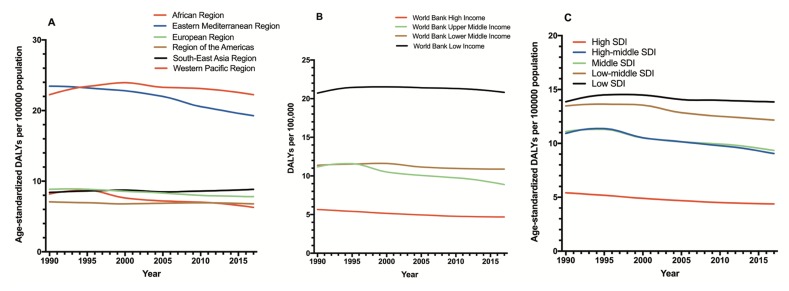
Global health burden of glaucoma vision loss in WHO regions and different levels of income or SDI regions. **Panel A.** WHO regions. **Panel B.** Different levels of income regions. **Panel C.** Different levels of SDI regions. WHO – World Health Organization, SDI – socio-demographic index.

According to the GBD2017, **Figure 2** shows the distribution of the disease burden of glaucoma in 2017. As expected, the countries with largest populations had the highest DALY numbers (**Figure 2****,** Panel A, D). After adjusting for population size, the DALY rate was highest in Tunisia (**Figure 2****,** Panel B, E). After adjusting for population size and age, Qatar had the greatest burden of glaucoma (**Figure 2****,** Panel C, F). The disease burden in WHO regions was also analysed. The age-standardized DALY rates in the African region was the highest of six regions, followed by the Eastern Mediterranean region. In addition, the age-standardized DALY rates in the African region and the Eastern Mediterranean region were higher than global average level (**Figure 3****,** Panel A). Each of the income level regions was also analysed. The age-standardized DALY rates were highest in low-income regions and lowest in high-income regions (**Figure 3****,** Panel B). The disease burden in each of the SDI level regions was analysed. As expected, the age-standardized DALY rates were highest in low SDI regions and lowest in high SDI regions (**Figure 3****,** Panel C).

### Country-level disease burden of glaucoma and other indictors

**Figure 4:**
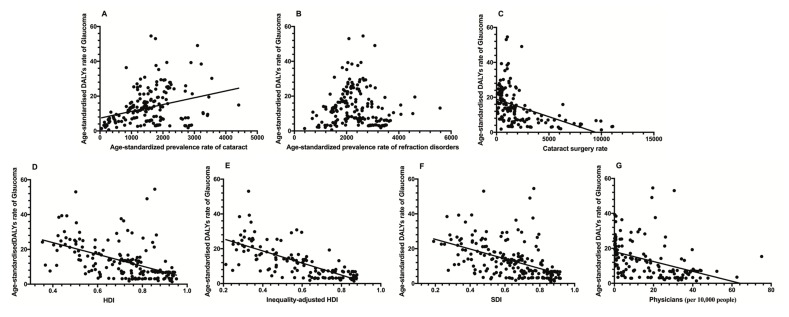
Relationship between the disease burden of glaucoma and other country-level indictors 2017. **Panel A.** Age-standardized prevalence rate of cataracts. **Panel B.** Age-standardized prevalence rate of refraction disorders. Panel C. Cataract surgery rate. **Panel D.** HDI. **Panel E.** Inequality-Adjusted HDI. **Panel F.** SDI. **Panel G.** Physicians’ rate. HDI – human development Index, SDI – socio-demographic index.

Linear regression analysis revealed that the country-level Age-standardized Prevalence Rate of Cataracts was significantly positively correlated with the age-standardized DALY rates of glaucoma while the country-level age-standardized prevalence rates of refraction disorders had no correlation with the age-standardized DALY rates of glaucoma. Conversely, the CRS, HDI, SDI, Inequality-Adjusted HDI, and physician’s rate had significantly negative correlations with the age-standardized DALY rates of glaucoma (**Table 2**, **Figure 4**).

**Table 2:** Linear regression analysis of the relationship between the country-level disease burden of glaucoma and the socioeconomic variables

	R square	*P*-value*	Coefficient	Lower bound	Upper bound
Age-standardized prevalence rate of cataracts	0.103	<0.001	0.004	0.002	0.006
Age-standardized prevalence rate of refraction disorders	-	0.307	-	-	-
CRS	0.202	<0.001	-0.002	-0.003	-0.001
HDI	0.244	<0.001	-33.817	-43.022	-24.612
Inequality-adjusted HDI	0.499	<0.001	-34.761	-40.735	-28.788
SDI	0.233	<0.001	-27.961	-35.169	-20.753
Physicians (per 10 000 people)	0.171	<0.001	-0.282	-0.382	-0.181
**Model 1†**	0.257	<0.001			
HDI			-22.168	-37.152	-7.183
CRS			-0.001	-0.002	-0.0001
**Model 2‡**	0.501	<0.001			
Mean years of schooling			-1.592	-2.431	-0.753
GNI per capita			0.0003	0.0002	0.0004
CRS			-0.001	-0.002	-0.001
Expected years of schooling			-1.085	-2.009	-0.161
**Model 3**§	0.520	<0.001	-	-	-
Inequality-adjusted HDI			-38.529	-46.276	-30.783

DALY – disability-adjusted life years, HDI – human development index, SDI – socio-demographic index, GNP – gross national income, CSR – cataract surgery rate

*P ≤ 0.05 were considered statistically significant.

†Model 1: Age-standardized Prevalence Rate of Cataracts, Age-standardized Prevalence Rate of Refraction Disorders, CRS, Physicians, HDI, and SDI.

‡Model 2: Age-standardized Prevalence Rate of Cataracts, Age-standardized Prevalence Rate of Refraction Disorders, CRS, Physicians, SDI, Mean Years of Schooling, GNI per Capita, Expected Years of Schooling, and Life Expectancy at Birth.

§Model 3: Age-standardized prevalence rate of cataracts, Age-standardized prevalence rate of refraction disorders, CRS, Physicians, SDI, and Inequality-Adjusted HDI.

Upon further analysis, the Inequality-adjusted HDI was the most influential indicator, accounting for 49.9% of global variations in age-standardized DALY rates of glaucoma. Stepwise multiple regression analysis was conducted to eliminate colinearity. Model 1 (which included the Age-standardized prevalence rate of cataracts, Age-standardized prevalence rate of refraction disorders, CRS, Physicians, HDI, and SDI) accounted for 25.7% of the global variations in the age-standardized DALY rates. The HDI and SDI significantly correlated with the disease burden of glaucoma. Model 2 (the four components of the HDI were used instead of the HDI) accounted for 50.1% of the global variations in the age-standardized DALY rates, and four indicators (Mean years of schooling, GNI per capita, CRS, and expected years of schooling) were significant. In Model 3, the Inequality-Adjusted HDI was used instead of the HDI. Only the Inequality-adjusted HDI was significant, and the model explained 52.0% of global variations in the age-standardized DALY rates (Table 2).

## DISCUSSION

This study provides an overview of the global distribution of the disease burden of glaucoma by year, age, gender, region, health care indictors, and socioeconomic levels. From 1990 to 2017, the world DALY rate of glaucoma increased by 81%. After adjusting for increases in population and ageing, the world age-standardized DALY rates decreased after 1995. The age and population-adjusted disease burden of glaucoma increased by age. Males had higher age-standardized DALY rates than females at the same age. Further research revealed that the disease burden of glaucoma was associated with Mean Years of Schooling, GNI per Capita, CRS, Expected Years of Schooling, and Inequality-Adjusted HDI, even after adjusting for the influence of cataracts and refraction disorders, socioeconomic factors, and health care indictors.

This study revealed that the prevalence of glaucoma increased in recent years. The number of people with glaucoma worldwide is expected to reach 111.8 million in 2040 [3]. Although the prevalence and DALY could not be compared directly, both types of information have implications for better understanding the effects of glaucoma as a disease. In fact, DALY is a more suitable indictor to measure the influence of a disease on the quality of life than the prevalence. Similar to the prevalence, this study’s results revealed that DALY due to glaucoma increased continuously from 1990 to 2017. The increase in DALY may be due to global population growth, ageing and increasing life expectancy, as DALY was closely associated with population number and life expectancy, and the ageing population leads to higher rates of onset of glaucoma [2]. These findings demonstrated that as the global population continues to increase and age, DALY due to glaucoma will continue to increase. Thus, rather than DALYs, DALY adjusted for age and population may be a better indicator for evaluating glaucoma service distribution since DALYs will be large if the population is large and ageing. After adjusting for age and population, the disease burden of glaucoma decreased over the past two decades. This may be associated with the contributions of the advances in glaucoma screening and diagnostic tools [26], such as automated perimetry and optical coherence tomography [27], which was developed in 1990 [28] and was first reported as a new tool for glaucoma diagnosis in 1995 [29,30]. This decrease may also be attributed to the advances in drugs, laser therapy, and surgery to control intraocular pressure [31-34]. Because early detection of glaucoma and effective control intraocular pressure can prevent further vision loss due to glaucoma, keeping vision loss minimal. These findings provide evidence toward the efforts of current actions and policies to reduce vision loss due to glaucoma as the DALY adjusted for age and population decreased over the past two decade. However, these findings also revealed that the advances in screening, diagnostic and treatment of glaucoma today still do not overcome the increasing of DALY due to glaucoma, which may be due to the increasing population sizes, ageing populations, extended life expectancy, and falling death rates. As there is no way to recover the impaired visual function due to glaucoma, as the population continues to grow and age, the next step to reduce DALYs due to glaucoma should be focus on finding ways to reduce incidence of glaucoma, (ie, gene therapy), recover the damaged neuron due to glaucoma, (ie, stem cell therapy [35]), and find more effective ways diagnose glaucoma at its early stage.

Our results revealed that regions with low income and low SDI have higher disease burden. This is consistent with previous studies. In a cross-sectional study of disease burden of glaucoma in 2017, there was a disproportionate distribution of the disease burden of glaucoma, which might have been influenced by many factors. A previous study from India demonstrated that socioeconomic status can influence the outcome of glaucoma, and urban residence (odds ratio (OR) = 0.6, *P* < 0.009), higher socioeconomic status (OR 0.3, *P* < 0.001), and higher awareness (approximately 50%, *P* < 0.007) can significantly lower the odds of having end-stage glaucoma [36]. In a study from China, males were more likely to have primary open angle glaucoma (POAG) (OR = 1.36, 95% CI = 1.17-1.59), but less likely to have primary angle closure glaucoma (PACG) (OR = 0.53, 95% CI = 0.46-0.60) compared to females. After adjusting for age and gender, urban residents was more likely to have POAG than rural residents [37]. A study from Taiwan showed that individuals who frequently utilized health care were more likely to be diagnosed with glaucoma early [38]. In a recent study, a systematic review of research demonstrated that low income may be associated with increased incidence of various sight-threatening conditions and may adversely affect access to specialised assessment and delivery of treatment [39]. Decreasing the disease burden in those regions should be a priority in future programs.

To further explore the factors associated with the disease burden of glaucoma, we analysed the country-level relationship of DALY rates and indictors. The country-level HDI was negatively correlated with the disease burden of glaucoma. The HDI can be used to rank countries according to key dimensions of human development, notably a long and healthy life, access to knowledge, and a decent standard of living. It has become an important indicator for comparisons of socioeconomic development across countries. When the four components were included in a model instead of the HDI, education was negatively correlated with the disease burden of glaucoma, which is consistent with previous country-level studies. In a study from the US, individuals with high education levels more frequently used eye care services [40]. Another study demonstrated that lower educational attainment may be associated with increased incidence of various sight-threatening conditions [39]. The educational level may influence the disease burden of glaucoma due to the following reasons: First, higher education usually means better awareness and knowledge of glaucoma, which may lead to early diagnosis of glaucoma [41]. Second, a higher education level usually leads to better adherence to recommended medications, which is key to controlling intraocular pressure [42]. Third, a higher education level leads to a greater possibility of a steady occupation, as well as higher income and coverage by medical insurance, enabling the cost of glaucoma treatments to be more affordable. This study revealed a positive association of GNI per capita and disease burden of glaucoma. GNI includes all the income earned by a country's residents and businesses, including any income earned abroad. Thus, GNI may not be an ideal indictor for evaluating the relationship between income and countries' disease burden. In many emerging markets, residents move to other countries where they can earn a better living and send money back to their families in their home county, but the countries’ DALY only calculates the disease burden of residents living within the country. This may cause bias when evaluating the relationship between income and countries' disease burden. However, more well-designed studies are needed to clarify the relationship between income and the disease burden of glaucoma.

The Inequality-Adjusted HDI was introduced in 2010 that adjusts the HDI for inequality in the distribution of each dimension in that population. In this study, the Inequality-Adjusted HDI demonstrated the most prominent component associated with the disease burden of glaucoma, which could explain 52.0% of the global variations in the age-standardized DALY rate. Compared to the HDI, it revealed that the less inequality in key dimensions of human development such as education and income, the more effective a country is at reducing the disease burden of glaucoma by development. This finding suggested that diminishing the inequality in resource distribution should be considered a priority for a country’s development.

The CRS is an indicator of eye care service delivery and estimating the burden of cataract disease. In this study, the CRS was negatively associated with the disease burden of glaucoma. The CRS may influence the disease burden of glaucoma due to the following reasons: First, both glaucoma and the medications to control intraocular pressure could accelerate the development of cataracts, and the vision loss of glaucoma patients may be due to the combined influence of glaucoma and cataracts. Second, recent studies revealed that cataract surgery can reduce intraocular pressure in patients with glaucoma [43-46]. This finding suggested that higher CRS may help reduce the disease burden of glaucoma.

This study has limitations. First, the accuracy of the disease burden information suffered from the limitations of the data sources. Second, because data were unavailable in a few countries, there are countries without corresponding data for all indictors. Notwithstanding the aforementioned limitations, the findings of this study could serve as an impetus for continued efforts to reduce the disease burden of glaucoma.

## CONCLUSIONS

In conclusion, this study provides contemporary estimates of the global disease burden of glaucoma from 1990 and 2017. The disease burden of glaucoma increased between 1990 and 2017 but decreased after adjusting for the age and population size over the past two decades. Older age and being male were associated with a higher burden of glaucoma. Higher education, higher CRS, and diminishing inequality in resource distribution may help reduce the disease burden of glaucoma. These findings can provide information for policymakers and could serve as an impetus for continued efforts to reduce the disease burden of glaucoma.
